# 679. Retrospective Analysis of Vancomycin and Fidaxomicin for the Treatment of Recurrent *Clostridioides difficile* Infection

**DOI:** 10.1093/ofid/ofad500.741

**Published:** 2023-11-27

**Authors:** Amy Fabian, Tyler Frantz, Rachana Patel, Karen Kier

**Affiliations:** University Hospitals St. John Medical Center, Westlake, Ohio; University Hospitals St. John Medical Center, Westlake, Ohio; University Hospitals St. John Medical Center, Westlake, Ohio; Ohio Northern University, Ada, Ohio

## Abstract

**Background:**

It is estimated that one out of every six patients with a *Clostridioides difficile* infection (CDI) will develop a recurrence. Guidelines recommend treatment with fidaxomicin rather than vancomycin standard course or tapered and pulsed regimens for the treatment of recurrent CDI (rCDI). The objective of this study is to evaluate clinical outcomes for three different pharmacological approaches for the treatment of rCDI.

**Methods:**

This is a multi-centered, retrospective cohort of patients who have had more than one episode of CDI who received either fidaxomicin, conventional vancomycin dosing, or vancomycin tapered and pulsed. Inclusion criteria are adult patients who have received at least one dose of oral vancomycin or fidaxomicin and had a positive stool assay for CDI from October 1st, 2021 to September 30th, 2022. Patients who presented with an initial CDI, were pregnant, experienced in-hospital death prior to completion of antibiotic, were less than 18 years old, and elected palliative or hospice care were excluded. The primary outcome is the proportion of patients who develop rCDI at eight weeks. Secondary outcomes include time to recurrence, proportion of patients prescribed guideline-preferred therapy, and the proportion of patients with rCDI or mortality at three months and six months from starting therapy for rCDI. Primary and secondary outcomes will be analyzed using ANOVA testing, and a multi-variable regression analysis was performed to identify significant risk factors for rCDI.

**Results:**

Among 71 patients, no difference was detected between fidaxomicin (n=11, 16%), vancomycin conventional dosing (n=48, 68%), and vancomycin tapered and pulsed (n=12, 17%) in the proportion of patients with rCDI at eight weeks (p = 0.335). A subgroup analysis of patients with infectious diseases (ID) consults (n=34, 47%) was performed, and they were more likely to be prescribed fidaxomicin (n=11, 32%; p = ≤ 0.001) and less likely to have rCDI (n=12, 17%; p = 0.008).

**Conclusion:**

Compliance with guideline preferred therapy for rCDI in the community hospital setting is low among non-ID providers. Further research should be conducted to determine the impact that an ID consult can have on clinical outcomes in patients with rCDI.

**Disclosures:**

**All Authors**: No reported disclosures

